# Leicester Cough Questionnaire: translation to Portuguese and cross-cultural adaptation for use in Brazil*

**DOI:** 10.1590/S1806-37132014000300003

**Published:** 2014

**Authors:** Manuela Brisot Felisbino, Leila John Marques Steidle, Michelle Gonçalves-Tavares, Marcia Margaret Menezes Pizzichini, Emilio Pizzichini

**Affiliations:** Universidade de São Paulo - USP, University of São Paulo - School of Medicine, São Paulo, Brazil; Department of Clinical Medicine, Universidade Federal de Santa Catarina - UFSC, Federal University of Santa Catarina - Florianópols, Brazil; Universidade do Sul de Santa Catarina - UNISUL, University of Southern Santa Catarina - Tubarão, Brazil; Department of Clinical Medicine, Universidade Federal de Santa Catarina - UFSC, Federal University of Santa Catarina - Florianópols, Brazil; Department of Clinical Medicine, Universidade Federal de Santa Catarina - UFSC, Federal University of Santa Catarina - Florianópols, Brazil

**Keywords:** Quality of life, Translations, Questionnaires, Cough

## Abstract

**Objective:**

To translate the Leicester Cough Questionnaire (LCQ) to Portuguese and adapt it for use in Brazil.

**Methods:**

Cross-cultural adaptation of a quality of life questionnaire requires a translated version that is conceptually equivalent to the original version and culturally acceptable in the target country. The protocol used consisted of the translation of the LCQ to Portuguese by three Brazilian translators who were fluent in English and its back-translation to English by another translator who was a native speaker of English and fluent in Portuguese. The back-translated version was evaluated by one of the authors of the original questionnaire in order to verify its equivalence. Later in the process, a provisional Portuguese-language version was thoroughly reviewed by an expert committee. In 10 patients with chronic cough, cognitive debriefing was carried out in order to test the understandability, clarity, and acceptability of the translated questionnaire in the target population. On that basis, the final Portuguese-language version of the LCQ was produced and approved by the committee.

**Results:**

Few items were questioned by the source author and revised by the committee of experts. During the cognitive debriefing phase, the Portuguese-language version of the LCQ proved to be well accepted and understood by all of the respondents, which demonstrates the robustness of the process of translation and cross-cultural adaptation.

**Conclusions:**

The final version of the LCQ adapted for use in Brazil was found to be easy to understand and easily applied.

## Introduction

Cough is one of the most common symptoms in clinical practice. Typically, cough is acute and self-limiting; however, in a significant proportion of patients, cough can present as an isolated chronic symptom.^(^ ^1^ ^)^ Such patients suffer considerable physical and psychological morbidity.^(^ ^2^ ^)^ Chronic cough is defined as any cough lasting more than eight weeks, with no concomitant clinical findings, and remaining without a definitive diagnosis after the initial clinical evaluation.^(^ ^3^ ^)^ Chief among the most common causes of cough are postnasal drip syndrome, cough variant asthma, gastroesophageal reflux disease, and eosinophilic bronchitis.^(^ ^4^ ^,^ ^5^ ^)^

The impact of symptoms over a given period of time can be quantified and standardized by means of generic quality-of-life questionnaires,^(^ ^6^ ^)^ or, more recently, by means of disease-specific questionnaires^(^ ^7^ ^,^ ^8^ ^)^ or questionnaires designed to assess a specific problem, such as chronic cough.^(^ ^9^ ^,^ ^10^ ^)^ Currently, there are two established questionnaires that assess quality of life in patients with cough: the Cough Quality-of-Life Questionnaire,^(^ ^9^ ^)^ developed by French et al.; and the Leicester Cough Questionnaire (LCQ),^(^ ^10^ ^)^ developed and validated by Birring et al. with the purpose of assessing this symptom and its impact on the health status of patients with chronic cough in a simple objective way. The LCQ can also be used to assess the temporal course of cough and monitor the response to treatment. The LCQ is self-administered and requires less than five minutes for completion. It comprises 19 items divided into three domains: physical (questions 1, 2, 3, 9, 10, 11, 14, and 15); psychological (questions 4, 5, 6, 12, 13, 16, and 17); and social (questions 7, 8, 18, and 19). Responses are given on a Likert-type scale ranging from 1 to 7 points. To calculate the LCQ score, the points assigned to each question in each domain must be aggregated and divided by the number of questions in each respective domain. The total score is the sum of each domain score and ranges from 3 to 21, with scores closer to 21 indicating better health status or a weaker influence of cough on patient quality of life.

Because the LCQ is a measure originally developed in the English language, it should be translated to the target language and adapted to the social and cultural circumstances of the target country; otherwise, another such measure should be developed.^(^ ^11^ ^)^ Therefore, cross-cultural adaptation of a psychometric measure is a complex process that requires a translated version that is conceptually equivalent to the original version and culturally acceptable in the target country. ^(^ ^12^ ^)^ Technical and semantic equivalence should be sought between the source and target versions in order to avoid misinterpretation of data in the future. Cross-cultural adaptation of a measure will be complete when the psychometric properties of the translated version have been evaluated.^(^ ^13^ ^)^

To date, no health-related quality-of-life measure for patients with chronic cough has been developed or validated for use in Brazil. Therefore, the purpose of the present study was to translate the LCQ^(^ ^10^ ^)^ to Portuguese and adapt it for use in Brazil.

## Methods

**Figure 1:**
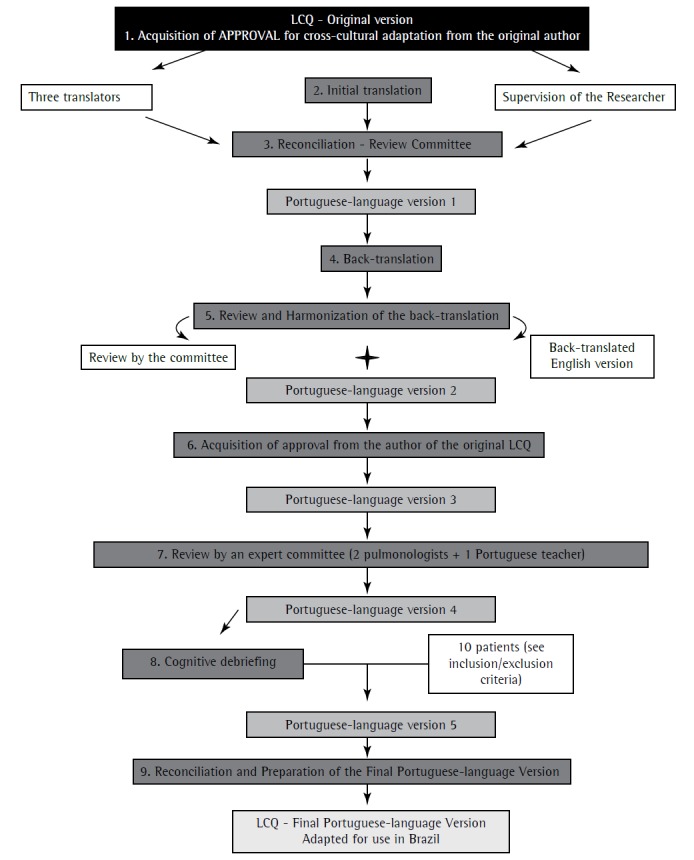
Summary of the process of translation and cross-cultural adaptation of the Leicester Cough Questionnaire (LCQ) for use in Brazil.

This was a methodological study involving the translation to Portuguese of a specific health-related quality-of-life measure for patients with chronic cough, the LCQ,^(^ ^10^ ^)^ and its cross-cultural adaptation for use in Brazil. The study was approved by the Human Research Ethics Committee of the *Universidade Federal de Santa Catarina* (UFSC, Federal University of Santa Catarina). The process of translation and cross-cultural adaptation of the LCQ was performed as described by Guillemin et al.^(^ ^14^ ^)^ and Wild et al.^(^ ^15^ ^))^ In Brazil, Tavares et al. used this methodology to translate an asthma control questionnaire to Portuguese and adapt it for use in Brazil.^ (^ ^16^ ^)^ Figure 1 illustrates each phase of the study.

The study sample intentionally consisted of 10 male and female patients over 18 years of age who were literate, had chronic cough, and were receiving no specific treatment. Those patients, recruited from the Pulmonology Outpatient Clinic of the UFSC University Hospital and from a private practice in respiratory medicine in the city of Florianópolis, Brazil, were invited to participate in the cognitive debriefing phase of the process of cross-cultural adaptation of the LCQ. This phase was used to assess the acceptability, clarity, and understandability of the translated adapted version.

For the present study, chronic cough was defined as cough lasting more than eight weeks and remaining without a definitive diagnosis after the initial clinical evaluation, which included chest X-ray as well as complete spirometry and bronchodilator response testing. We excluded smokers, former smokers, patients with other lung diseases (cystic fibrosis, COPD, pneumonia, etc.), patients with severe diseases of other body systems, and patients on medications that could confound the results. Since the present study does not permit a statistical analysis, the data are reported as absolute numbers and proportions, as means and standard deviations, or as medians and interquartile ranges.

The phases of the cross-cultural adaptation process were performed strictly in accordance with internationally accepted guidelines^(^ ^14^ ^)^: acquisition of permission for cross-cultural adaptation and of the rights of use of the LCQ from the developer of the questionnaire; translation of the LCQ from English to Portuguese; reconciliation; back-translation; review and harmonization of the back-translation; acquisition of approval from the developer of the LCQ; review of the Portuguese-language version of the LCQ by experts; cognitive debriefing; and reconciliation and preparation of the final version.

In the English-to-Portuguese translation phase, three Brazilian translators who were fluent in English independently translated the LCQ. Subsequently, a review committee met to produce a first Portuguese-language version. This first version was back-translated to English by another translator who was a native speaker of English and fluent in Portuguese. The back-translation was then reviewed by the committee, which produced a back-translated English version and a matching Portuguese-language version of the LCQ. The back-translated version was sent to the author of the original LCQ for evaluation, and, once approved, its matching version was used to produce a third Portuguese-language version of the LCQ. This third version was reviewed by an expert committee, which consisted of two bilingual pulmonologists and a Portuguese teacher, and, subsequently, a fourth Portuguese-language version of the LCQ was produced. This fourth version was used in the cognitive debriefing phase, at the end of which a fifth version was produced. After reconciliation, the final Portuguese-language version of the LCQ was produced (Figure 1).

The purpose of cognitive debriefing was to identify problematic questions on the questionnaire and offer solutions to make such questions easier to understand. To that end, ten participants with chronic cough who showed good comprehension and language skills were interviewed. The cognitive debriefing process consisted of testing the understandability, clarity, and acceptability of the translated questionnaire in the target population. In this phase, individuals who met the inclusion criteria were consecutively scheduled for a single visit to the study site. During this visit, the study was explained in detail, and individuals who agreed to participate gave written informed consent. In addition, we collected demographic data and specific data on current and previous history of cough, duration and characteristics of cough, associated symptoms, final diagnosis (if defined), smoking history, and comorbidities. The questionnaire was administered to each participant by the principal investigator. Individuals were informed that they should not worry about the accuracy of their responses, but rather just report what they understood, the difficulty of each question or statement on the questionnaire, and their level of acceptance of the questionnaire. At the end, individuals were asked to make a general open comment about the questionnaire so that its overall acceptability, understandability, and clarity could be assessed. All comments were recorded on a specific form.

Finally, in the reconciliation phase, the review committee and the expert committee met to produce the final Portuguese-language version of the LCQ. To that end, the latest provisional version of the measure was analyzed item by item. The cognitive debriefing findings were discussed, and the relevant changes were made. Therefore, the final Portuguese-language version of the measure was produced.

## Results

Of the ten patients interviewed in the cognitive debriefing phase, seven were female. All were White, were nonsmokers, and resided in the greater metropolitan area of Florianópolis, Brazil. Patient age ranged from 23 to 72 years, and patient educational level ranged from elementary school to college. Most patients had dry cough, which was associated with other symptoms, such as nasal obstruction, sneezing, and odynophagia, in 40% of the cases. Only two patients had no comorbidities, and the most common comorbidities were systemic arterial hypertension, type 2 diabetes mellitus, dyslipidemia, hypothyroidism, allergic rhinitis, and depression (Table 1).

**Table 1:** Distribution of patients by demographic and disease-specific characteristics.a

Characteristic	Result
Age, years^b^	52.1 ± 14.6
Female gender	7 (70)
High school diploma or less	5 (50)
Duration of cough, months^c^	90 (10-198)
Dry cough	7 (70)
Presence of associated symptoms^d^	4 (40)
Presence of comorbidities^e^	8 (80)

a Values expressed as n (%), except where otherwise indicated

b Value expressed as mean ± SD

c Value expressed as median (interquartile range)

d Odynophagia (in 10%); nasal obstruction (in 20%); and sneezing (in 10%)

e Allergic rhinitis (in 40%); systemic arterial hypertension (in 30%); dyslipidemia (in 30%); depression (in 20%); diabetes mellitus (in 10%); and hypothyroidism (in 10%)

Half of the patients interviewed were still undergoing diagnostic evaluation. For the remaining patients, one or more causes of cough had been found (Table 2).

**Table 2:** Distribution of patients by final diagnosis.a

Diagnosis	Result
Under investigation	5 (50)
Chronic sinusitis	3 (30)
Eosinophilic bronchitis	3 (30)
Gastroesophageal reflux disease	1 (10)
Cough variant asthma	1 (10)

a Values expressed as n (%). Note: Any given patient may have more than one diagnosis.

In the phases of translation and back-translation, no questions or corrections were raised. However, in the phase of acquisition of approval from the author of the original LCQ, some items on the back-translated version were in part questioned by him because they showed a slight difference in wording. However, since the concept was preserved, no changes were made. The following items were questioned: "by sputum (phlegm) production when you cough?", which was back-translated as "by any phlegm you've coughed up?"; and "with the overall enjoyment of my life", which was back-translated as "with the enjoyment of my life".

The review performed by the expert committee indicated some grammatical errors and offered conceptual suggestions, all of which are described in Table 3. In addition, the questionnaire formatting was modified: the Likert-type scale with response choices arranged in horizontal sequence was placed within a single-row, seven-column table (Appendix 1; available in the online version of the Brazilian Journal of Pulmonology; http://www.jornaldepneumologia.com.br/imagebank/images/jbp_v40n3_anexo.pdf).

**Table 3:** Changes made after the review by the expert committee.

LCQ – Portuguese-language version 3	LCQ – Portuguese-language version 4
“Elaborado”	“Desenvolvido”
“Responda circulando a resposta”	“Circule o número da resposta”
“O mais honestamente possível”	“Da maneira mais honesta possível”
“Como consequência”	“Em consequência”
“Esteve incomodado”	“Se incomodou”
“Esteve cansado”	“Se cansou”
“Me fez sentir ansioso”	“Me deixou ansioso”
“No aproveitamento da minha vida”	“No prazer de aproveitar minha vida”
“Saturado”	“Farto”
“Ficou preocupado”	“Se preocupou”
“Incomodou”	“Aborreceu”
“Responder este questionário”	“Responder a este questionário”

LCQ : Leicester Cough Questionnaire.

In the cognitive debriefing phase, three questions produced understandability difficulties. In addition, the title of the questionnaire was a source of difficulty for nearly half of the respondents. Therefore, in the final reconciliation phase, in which the review committee and the expert committee met, it was unanimously agreed that changes should be made to the title and to two of the questions. Table 4 shows the changes made after cognitive debriefing. The final version of the document incorporated those changes, as shown in Appendix 1.

**Table 4:** Changes made after the cognitive debriefing process.

LCQ – Portuguese-language version 4	LCQ – Portuguese-language version 5
“Questionário de Tosse Leicester”	“Questionário de Leicester sobre Tosse”
“Nas últimas 2 semanas, minha tosse me fez sentir farto (cheio).”	“Nas últimas 2 semanas, minha tosse me fez sentir de “saco cheio”
“Nas últimas 2 semanas, você teve muita energia?”	“Nas últimas 2 semanas, mesmo com sua tosse, você teve muita energia?”

LCQ : Leicester Cough Questionnaire.

## Discussion

In the present study, a health-related quality-of-life measure for patients with chronic cough was translated to Portuguese and adapted for use in Brazil. The original version of the LCQ was developed primarily to assess patients in English, and, to date, only a Dutch-language version has been produced and validated.^(^ ^17^ ^)^ Cross-cultural adaptation is relevant because, currently, there is no other quality-of-life measure for patients with chronic cough in Brazil. The decision to culturally adapt the LCQ, rather than to develop a new measure, was based on the fact that the adaptation of a previously described and validated measure, which has been translated and validated to other languages, makes it possible to compare results across studies conducted in different countries. In addition, this is a current trend that aims to facilitate the use of such a measure in international multicenter studies and has boosted the translation and cross-cultural adaptation of several generic and specific instruments to several languages.^(^ ^18^ ^,^ ^19^ ^)^ Furthermore, the development of a new questionnaire would be a more laborious, time-consuming, and costly process.

Kalpaklioglu et al.^(^ ^20^ ^)^ compared the LCQ with the Cough Quality-of-Life Questionnaire and showed that there is a significant correlation between the measurements of the two questionnaires. The present study aimed to translate and culturally adapt the LCQ because it is a careful questionnaire, which consists of well-formulated questions and is structured by domains. The methodology used in the development of the LCQ^(^ ^10^ ^)^ ensures proper validation of content. In addition, the LCQ is valid and reproducible,^(^ ^10^ ^)^ as well as being discriminative^(^ ^21^ ^)^ and responsive to longitudinal changes.^(^ ^10^ ^)^ Several studies have successfully used the LCQ to assess the response to several therapies for cough, as has been shown by Ryan et al.^(^ ^22^ ^)^ for gabapentin therapy for refractory chronic cough and by Patel et al.^(^ ^23^ ^)^ for cough-suppression physiotherapy. Therefore, guidelines on the management of chronic cough describe the LCQ as an important tool for quantification of cough and assessment of patient quality of life,^(^ ^24^ ^-^ ^26^ ^)^ since there are few objective and well-validated instruments for quantification of cough. In more recent studies, the LCQ has been validated for assessment of chronic cough in the context of specific diseases^(^ ^27^ ^,^ ^28^ ^)^ and for use in acute cough.^(^ ^29^ ^)^

One factor that ensures the applicability of the LCQ in Brazil is the methodology used in the process of translation and cross-cultural adaptation of the questionnaire, which has been shown to preserve the sensitivity of the measure,^(^ ^14^ ^)^ as well as promoting an appropriate level of equivalence between the versions. In addition, it is known that the internal structure, semantics, and psychometric characteristics of a measure may change when this measure is translated to another language. This is more common if the process of cross-cultural equivalence is not performed correctly. The need to take into account cultural influences on health and disease is increasingly being recognized in multicenter and multinational studies. The purpose of adapting a quality-of-life measure is to obtain health measurements that are appropriate and valid in different cultural groups. This means developing a measure that is conceptually equivalent in different cultures.^(^ ^30^ ^)^

In the present study, the difficulties encountered in the translation phase resulted from the need to produce a conceptual translation. There were no difficulties in translating words referring to symptoms, physical activities, or activities of daily living. However, some English-language idioms and phrases, such as "fed up" and "overall enjoyment", were a matter of review and discussion. In addition, there was a need to adjust the verb tense so that the addressed situation made sense in Portuguese. In the phase of acquisition of approval from the original author, only two items were questioned by him as to differences in the literal translation. However, since, according to the original author himself, conceptual equivalence was preserved, no changes were needed. Once the back-translated version was approved, an expert committee met to evaluate its matching Portuguese-language version in order to detect errors, make suggestions, and analyze content and structure. In this phase, it is of particular value that the expert committee include bilingual members.^(^ ^14^ ^)^

The first modification was to the questionnaire formatting. The original version uses a Likert-type scale with response choices arranged in horizontal sequence. In the Portuguese-language version, the same Likert-type scale was placed within a single-row, seven-column table. The modification made it easier to visualize all response choices. In order to achieve semantic, conceptual, and idiomatic equivalence, some expressions, words, prepositions, and verb tenses were changed. The difficulty lies in the fact that some English-language expressions have no literal equivalent in Portuguese, and, in such cases, conceptual equivalence is sought. Corrections of grammatical errors were made by the Portuguese-language expert, and the questionnaire version intended for use in the cognitive debriefing phase was then produced.

Cognitive debriefing is an essential phase in the cross-cultural adaptation process, because even a detailed methodological process does not ensure equivalence between target and source versions.^(^ ^14^ ^)^ The questionnaire was administered to ten participants in order to determine its acceptability, clarity, and understandability. Although the participants had varied educational levels, no significant difficulties that would prevent them from understanding the questionnaire were identified. This demonstrates that the measure produced can be administered to individuals from various socio-cultural classes. To ensure that the entire translation was easy to understand, cognitive debriefing involved an item-by-item review, rather than a random sample review. An analysis of the responses given during the cognitive debriefing process showed that few items needed to be revised because of understandability difficulties. This finding is of great relevance because it shows the robustness of the process of translation and cross-cultural adaptation. Therefore, the final version was produced after changes, which were unanimously agreed by the review committee and the expert committee, were made to three items, among which was the title of the questionnaire.

The respondents' comments on the questionnaire were very positive. All stated that, in general, the questionnaire was clear, easy to understand, and easy to answer, with simple and quick-to-follow instructions. In addition, the questionnaire was considered to be significantly relevant in the evaluation of chronic cough, being well adapted to that condition and covering its various aspects in detail.

In conclusion, the LCQ has been translated to Portuguese and adapted for use in Brazil. The final Portuguese-language version of the questionnaire, designated Questionário de Leicester sobre Tosse Crônica, was found to be easy to understand and easily applied, as well as being a single measure of health-related quality-of-life variables in patients with chronic cough.
