# Supplementary material for: Leicester Cough Questionnaire: translation to Portuguese and cross-cultural adaptation for use in Brazil
Source: J Bras Pneumol. 2014 May-Jun;40(3):213–21. doi: 10.1590/S1806-37132014000300003 (PMC4109192; doi:10.1590/S1806-37132014000300003)
Supplement: Translation

# Translation: Questionário de Leicester sobre tosse crônica: tradução e adaptação cultural para a língua portuguesa falada no Brasil*

**Authors:** Manuela Brisot Felisbino, Leila John Marques Steidle, Michelle Gonçalves-Tavares, Marcia Margaret Menezes Pizzichini, Emilio Pizzichini

**Affiliations:** Faculdade de Medicina, Universidade de São Paulo - USP - São Paulo (SP) Brasil; Departamento de Clínica Médica, Universidade Federal de Santa Catarina - UFSC - Florianópolis (SC) Brasil; Universidade do Sul de Santa Catarina - UNISUL - Tubarão (SC) Brasil; Departamento de Clínica Médica, Universidade Federal de Santa Catarina - UFSC - Florianópolis (SC) Brasil; Departamento de Clínica Médica, Universidade Federal de Santa Catarina - UFSC - Florianópolis (SC) Brasil

**Keywords:** Qualidade de vida, Traduções, Questionários, Tosse

## Abstract

**Objetivo:**

Traduzir e adaptar culturalmente o *Leicester Cough Questionnaire* (LCQ) para a língua portuguesa falada no Brasil.

**Métodos:**

A adaptação cultural de um questionário de qualidade de vida envolve a tradução conceitualmente equivalente à versão original e culturalmente aceitável ao país em que será utilizado. O protocolo aplicado consistiu na tradução do LCQ para a língua portuguesa por três tradutores brasileiros com fluência na língua inglesa e sua retradução para a língua original por um tradutor nascido em um país de língua inglesa e com fluência na língua portuguesa. A versão retraduzida foi avaliada por um dos autores do questionário original para assegurar sua equivalência e, posteriormente, o questionário foi revisado por um comitê de especialistas que realizou ampla revisão do instrumento. O desdobramento cognitivo consistiu em testar a compreensão, clareza e aceitabilidade do questionário traduzido na população alvo, aplicando-o em dez pacientes portadores de tosse crônica. Com base nisso, foi realizada a formulação da versão brasileira final do LCQ após sua aprovação pelo comitê.

**Resultados:**

Poucos itens foram questionados pelo autor da versão original e revistos pelo comitê de especialistas. A versão portuguesa do LCQ apresentou boa aceitabilidade e compreensão por todos os entrevistados no desdobramento cognitivo, demonstrando a robustez do processo de tradução e adaptação cultural.

**Conclusões:**

A versão final traduzida e adaptada para uso no Brasil mostrou ser de fácil compreensão e aplicação.

## Introdução

A tosse se apresenta como um dos sintomas mais comuns na prática clínica. Habitualmente a tosse é aguda e autolimitada; no entanto, em uma proporção importante de pacientes, a tosse pode se apresentar como um sintoma crônico isolado.^(^ ^1^ ^)^ Esses pacientes sofrem uma considerável morbidade física e psicológica.^(^ ^2^ ^)^ Tosse crônica é definida como toda tosse com duração superior a oito semanas, sem outros achados clínicos concomitantes, permanecendo sem diagnóstico definitivo após a avaliação clínica inicial.^(^ ^3^ ^)^ Dentre as causas mais frequentes de tosse crônica, destacam-se a síndrome do gotejamento pós-nasal, tosse variante da asma, doença do refluxo gastroesofágico e bronquite eosinofílica.^(^ ^4^ ^,^ ^5^ ^)^

A quantificação e a padronização do impacto dos sintomas em um determinado período de tempo podem ser obtidas através de questionários de qualidade de vida genéricos,^(^ ^6^ ^)^ ou, mais recentemente, através de questionários especificamente construídos para uma determinada doença^(^ ^7^ ^,^ ^8^ ^)^ ou ainda para um determinado problema, como o caso da tosse crônica.^(^ ^9^ ^,^ ^10^ ^)^ Atualmente existem dois questionários válidos que avaliam a qualidade de vida em pacientes portadores de tosse: o *Cough Quality-of-Life Questionnaire*,^(^ ^9^ ^)^ desenvolvido por French et al., e o *Leicester Cough Questionnaire* (LCQ),^(^ ^10^ ^)^ desenvolvido e validado por Birring et al. com o objetivo de avaliar de forma objetiva e simples esse sintoma e seu impacto no estado de saúde dos portadores de tosse crônica. O LCQ pode, também, ser usado na avaliação temporal da evolução da tosse e no acompanhamento da resposta ao tratamento. O LCQ é autoaplicativo e requer menos de cinco minutos para ser concluído. É composto por 19 itens subdivididos em três domínios: físico (perguntas 1, 2, 3, 9, 10, 11, 14 e 15), psicológico (perguntas 4, 5, 6, 12, 13, 16 e 17) e social (perguntas 7, 8, 18 e 19). As respostas são quantificadas pelo paciente em uma escala Likert que varia de 1 a 7 pontos. Para o cálculo do LCQ, deve-se realizar uma soma da pontuação das perguntas de cada domínio. Divide-se esse valor pelo número de perguntas do respectivo domínio. O escore total é o resultado da adição dos escores de cada domínio e varia de 3 a 21, sendo que uma pontuação mais próxima de 21 indica um melhor estado de saúde ou uma menor influência da tosse na qualidade de vida do paciente.

Por se tratar de um instrumento desenvolvido originalmente na língua inglesa, o LCQ deve ser traduzido e adaptado às circunstâncias sociais e culturais do local onde se pretende utilizá-lo, caso contrário um novo instrumento deveria ser desenvolvido com esse objetivo.^(^ ^11^ ^)^ Dessa forma, a adaptação cultural de um instrumento psicométrico é um processo complexo que envolve sua tradução conceitualmente equivalente ao original e culturalmente aceitável ao país no qual o questionário será utilizado.^(^ ^12^ ^)^ Devem-se buscar equivalências técnicas e semânticas entre as versões fonte e alvo para se evitar futuros erros de interpretação dos dados. A adaptação cultural de um instrumento estará completa quando as qualidades psicométricas do instrumento traduzido forem estudadas.^(^ ^13^ ^)^

Até o presente, nenhum instrumento de avaliação da qualidade de vida e estado de saúde em pacientes portadores de tosse crônica foi elaborado ou validado para uso no Brasil. Portanto, o presente estudo teve como objetivo a tradução e a adaptação cultural do LCQ^(^ ^10^ ^)^ para a língua portuguesa falada no Brasil.

## Métodos

Este é um estudo metodológico que envolve a tradução e a adaptação cultural, para a língua portuguesa falada no Brasil, de um instrumento específico para medir a qualidade de vida relacionada ao estado de saúde em pacientes portadores de tosse crônica, o LCQ.^(^ ^10^ ^)^ O estudo foi aprovado pelo Comitê de Ética em Pesquisa com Seres Humanos da Universidade Federal de Santa Catarina (UFSC). A tradução e a adaptação cultural do LCQ seguiram os pressupostos publicados por Guillemin et al.^(^ ^14^ ^)^ e Wild et al.^(^ ^15^ ^)^ No Brasil, Tavares et al. utilizaram essa metodologia para a adaptação cultural de um questionário de controle de asma para a língua portuguesa falada no Brasil. ^(^ ^16^ ^)^ A Figura 1 especifica cada etapa da pesquisa.

**Figura 1:** Súmula do processo de adaptação transcultural do Leicester Cough Questionnaire (LCQ) para a língua portuguesa falada no Brasil.

A população do estudo foi intencionalmente composta por dez pacientes de ambos os sexos, alfabetizados, com idade superior a 18 anos, portadores de tosse crônica e sem uso de tratamento específico. Esses pacientes, selecionados do Ambulatório de Pneumologia do Hospital Universitário da UFSC e de uma clínica privada de medicina respiratória de Florianópolis (SC), foram convidados a participar da etapa de desdobramento cognitivo do processo de adaptação cultural do LCQ. Essa etapa foi utilizada para demonstrar a aceitabilidade, clareza e compreensão do questionário traduzido e adaptado.

Para o presente estudo, tosse crônica foi definida como a tosse com duração maior do que oito semanas, permanecendo sem diagnóstico definitivo após a avaliação clínica inicial, que incluiu radiografia de tórax e espirometria completa com teste de resposta a um broncodilatador. Foram excluídos do estudo fumantes ou ex-fumantes, portadores de outras doenças pulmonares (fibrose cística, DPOC, pneumonia, etc.), portadores de doenças graves de outros aparelhos, ou aqueles em uso de medicações que pudessem confundir os resultados. Como o presente estudo não comporta uma análise estatística, os dados foram relatados como números absolutos e proporções, como médias e desvios-padrão ou como medianas e intervalos interquartílicos.

As etapas realizadas para o processo de adaptação cultural foram seguidas rigorosamente conforme sugestões internacionalmente aceitas^(^ ^14^ ^)^: autorização e cessão de direitos de uso do LCQ do idealizador do questionário; tradução do LCQ da língua inglesa para a língua portuguesa; reconciliação; retradução; revisão e harmonização da retradução; aprovação do idealizador do LCQ; revisão da versão em português do LCQ por especialistas; desdobramento cognitivo; e reconciliação para a elaboração da versão final.

Na etapa de tradução da língua inglesa para a língua portuguesa, três tradutores brasileiros com fluência na língua inglesa realizaram de forma independente a tradução do LCQ. Posteriormente, reuniu-se o comitê revisor para a confecção da primeira versão na língua portuguesa. Essa versão foi retraduzida para a língua inglesa por um tradutor nascido em país de língua inglesa e com fluência na língua portuguesa. Foi então realizada uma nova revisão pelo comitê revisor e elaborada a segunda versão do LCQ para a língua portuguesa. A versão retraduzida para o inglês foi enviada ao autor do LCQ original para sua avaliação e, após sua aprovação, foi elaborada a terceira versão portuguesa do LCQ. Essa versão foi revisada por um comitê de especialistas, composto por dois pneumologistas bilíngues e um professor de língua portuguesa, sendo posteriormente elaborada a quarta versão do LCQ em português. Com essa versão foi realizado o desdobramento cognitivo e, ao final dessa etapa, foi elaborada a quinta versão. Após a reconciliação, foi elaborada a versão final do LCQ em língua portuguesa (Figura 1).

O desdobramento cognitivo teve como objetivo identificar questões problemáticas do questionário e oferecer soluções para facilitar seu entendimento. Para tanto, foram entrevistados dez participantes portadores de tosse crônica que apresentassem boas condições de compreensão e linguagem. O desdobramento cognitivo consistiu no processo de testar, na população alvo, a compreensão, a clareza e a aceitabilidade do questionário traduzido. Nessa etapa, os participantes que preencheram os critérios de inclusão foram consecutivamente agendados para uma única visita ao local do estudo. Nessa visita, o estudo foi detalhadamente explicado, e os indivíduos que concordaram em participar assinaram o consentimento livre e informado. Durante a visita também foram coletados dados demográficos e específicos quanto à história da doença atual e pregressa, duração e características da tosse, sintomas associados, diagnóstico final (se definido), história de tabagismo e comorbidades. O questionário foi aplicado pelo pesquisador principal a cada participante. Os indivíduos foram informados de que não deveriam se preocupar com a acurácia das respostas, mas apenas mostrar o que compreenderam, quais as dificuldades de cada pergunta ou afirmação do questionário e sua aceitação ao mesmo. Ao final, foi solicitado um comentário aberto geral sobre o questionário para avaliar sua aceitabilidade, entendimento e clareza de forma global. Todos os comentários foram devidamente registrados em um formulário específico.

Ao final, na etapa de reconciliação, reuniram-se para produzir a versão final do LCQ na língua portuguesa, o comitê revisor e de especialistas, onde o instrumento foi revisto item por item. Foram discutidos os achados do desdobramento cognitivo e incorporadas as modificações pertinentes, visando à confecção da versão final do questionário. Dessa forma, foi elaborada a versão final do instrumento adaptado para a língua portuguesa falada no Brasil.

## Resultados

Dos dez pacientes entrevistados para a etapa do desdobramento cognitivo, sete eram do sexo feminino. Todos eram da raça branca, não tabagistas e procedentes da grande Florianópolis. A faixa etária variou de 23 a 72 anos, e a escolaridade incluiu participantes entre o ensino fundamental e o ensino superior. A tosse apresentava característica seca na maioria dos pacientes, estando associada a outros sintomas, tais como obstrução nasal, espirros e odinofagia, em 40% dos casos. Somente dois pacientes não apresentaram comorbidades, sendo as mais relevantes hipertensão arterial sistêmica, diabetes mellitus tipo 2, dislipidemia, hipotireoidismo, rinite alérgica e depressão (Tabela 1).

**Tabela 1:** Distribuição dos pacientes segundo características demográficas e específicas.a

Características	Resultados
Idade, anos^b^	52,1 ± 14,6
Sexo feminino	7 (70)
Escolaridade até ensino médio	5 (50)
Duração da tosse, meses^c^	90 (10-198)
Tosse seca	7 (70)
Presença de sintomas associados^d^	4 (40)
Presença de comorbidades^e^	8 (80)

a Valores expressos em n (%), exceto onde indicado

b Valor expresso em média ± dp

c Valor expresso em mediana (intervalo interquartílico)

d Odinofagia (10%), obstrução nasal (20%) e espirros (10%)

e Rinite alérgica (40%), hipertensão arterial sistêmica (30%), dislipidemia (30%), depressão (20%), diabetes mellitus (10%) e hipotireoidismo (10%).

Metade dos pacientes entrevistados ainda estava sob investigação diagnóstica. Para os demais pacientes, uma ou mais causas para a tosse haviam sido encontradas (Tabela 2).

**Tabela 2:** Distribuição dos pacientes segundo o diagnóstico final.a

Diagnóstico	Resultados
Em investigação	5 (50)
Sinusite crônica	3 (30)
Bronquite eosinofílica	3 (30)
Doença do refluxo gastroesofágico	1 (10)
Tosse variante da asma	1 (10)

a Valores expressos em n (%). Obs.: o mesmo paciente pode ter mais de um diagnóstico.

As etapas de tradução e retradução do LCQ não suscitaram dúvidas ou correções. Entretanto, na etapa da aprovação pelo autor, parte de dois itens foram questionados pelo autor da versão original do LCQ por apresentarem uma pequena diferença na formulação da versão retraduzida. Contudo, como o conceito foi preservado, essas foram mantidas. Os itens questionados foram os seguintes: "*by sputum (phlegm) production when you cough?*" que foi retraduzido como "*by any phlegm you've coughed up?*" e "*with the overall enjoyment of my life*", retraduzido como "*with the enjoyment of my life*".

A revisão realizada pelo comitê de especialistas apontou alguns erros gramaticais e sugestões conceituais, descritos na Tabela 3. Foi alterada também a formatação do questionário, que dispunha a escala Likert em posição sequencial horizontal para uma tabela horizontal com sete colunas internas em linha única, nas quais todas as opções foram dispostas em um contínuo crescente (Anexo 1; disponível na versão on-line do Jornal Brasileiro de Pneumologia; http://www.jornaldepneumologia.com.br/imagebank/images/jbp_v40n3_anexo.pdf).

**Tabela 3:** Itens alterados após a revisão do comitê de especialistas.

LCQ – versão 3/português	LCQ – versão 4/português
“Elaborado”	“Desenvolvido”
“Responda circulando a resposta”	“Circule o número da resposta”
“O mais honestamente possível”	“Da maneira mais honesta possível”
“Como consequência”	“Em consequência”
“Esteve incomodado”	“Se incomodou”
“Esteve cansado”	“Se cansou”
“Me fez sentir ansioso”	“Me deixou ansioso”
“No aproveitamento da minha vida”	“No prazer de aproveitar minha vida”
“Saturado”	“Farto”
“Ficou preocupado”	“Se preocupou”
“Incomodou”	“Aborreceu”
“Responder este questionário”	“Responder a este questionário”

LCQ : *Leicester Cough Questionnaire*.

Na etapa de desdobramento cognitivo, três perguntas geraram dificuldades de compreensão, sendo que o título do questionário também foi motivo de dúvida por quase metade dos entrevistados. Assim, na etapa final de reconciliação, na qual se reuniram o comitê revisor e o de especialistas, optou-se, por unanimidade, pela modificação do título do questionário e de duas das perguntas. A Tabela 4 apresenta os itens modificados após o desdobramento cognitivo. A versão final do documento incorporou essas alterações conforme mostra o Anexo 1.

**Tabela 4:** Itens alterados após a realização do processo de desdobramento cognitivo.

LCQ – versão 4/português	LCQ – versão 5/português
“Questionário de Tosse Leicester”	“Questionário de Leicester sobre Tosse Crônica”
“Nas últimas 2 semanas, minha tosse me fez sentir farto (cheio).”	“Nas últimas 2 semanas, minha tosse me fez sentir de “saco cheio”
“Nas últimas 2 semanas, você teve muita energia?”	“Nas últimas 2 semanas, mesmo com sua tosse, você teve muita energia?”

LCQ : *Leicester Cough Questionnaire*.

## Discussão

No presente estudo, traduzimos e adaptamos culturalmente para a língua portuguesa falada no Brasil um instrumento para a avaliação da qualidade de vida relacionada ao estado de saúde em pacientes portadores de tosse crônica. A versão original do LCQ foi desenvolvida primariamente para a avaliação de tosse crônica na língua inglesa e, até o presente momento, apenas a versão para o holandês foi devidamente traduzida e validada. ^(^ ^17^ ^)^ A adaptação cultural é relevante porque, até o presente, não se dispõe de nenhum outro instrumento de avaliação de qualidade de vida nesse grupo de pacientes no Brasil. A escolha de adaptar culturalmente o LCQ, ao invés de se desenvolver um novo, foi fundamentada no fato de que a adaptação de um instrumento já previamente descrito e validado em outras línguas possibilita a comparação de resultados entre estudos realizados em diferentes países. Isso é uma tendência atual também para facilitar sua utilização em estudos multicêntricos internacionais e tem impulsionado a tradução e a adaptação cultural de diversos instrumentos genéricos e específicos em diversas línguas.^(^ ^18^ ^,^ ^19^ ^)^ Ademais, a elaboração de um novo questionário seria um processo mais laborioso, demorado e dispendioso.

Kalpaklioglu et al.^(^ ^20^ ^)^ compararam o LCQ e o *Cough Quality-of-Life Questionnaire* e mostraram uma correlação significativa entre as medidas dos dois questionários. O presente trabalho se propôs a traduzir e a adaptar culturalmente o LCQ porque se trata de um questionário criterioso, com perguntas bem formuladas e estruturado por domínios. A metodologia utilizada no desenvolvimento do LCQ^(^ ^10^ ^)^ assegura uma boa validação de conteúdo, sendo esse questionário válido e reprodutível,^(^ ^10^ ^)^ assim como discriminativo^(^ ^21^ ^)^ e responsivo a mudanças longitudinais.^(^ ^10^ ^)^ Diversos estudos vêm utilizando o LCQ com sucesso para a avaliação da resposta a diversos tratamentos de tosse, como mostrado por Ryan et al.^(^ ^22^ ^)^ no uso de gabapentina para tosse crônica refratária e por Patel et al.^(^ ^23^ ^)^ com o uso de fisioterapia supressora de tosse. Por isso, diretrizes sobre o manejo da tosse crônica descrevem o LCQ como uma alternativa importante para quantificar o sintoma e avaliar a qualidade de vida nesses pacientes,^(^ ^24^ ^-^ ^26^ ^)^ já que existem poucos instrumentos objetivos e bem validados para a sua quantificação. Em estudos mais recentes, o LCQ vem sendo inclusive validado para a avaliação da tosse crônica no contexto de doenças específicas^(^ ^27^ ^,^ ^28^ ^)^ e para o uso na tosse aguda.^(^ ^29^ ^)^

Um dos aspectos que garante a aplicabilidade do LCQ em nosso meio é a metodologia utilizada no processo de tradução e adaptação cultural desse questionário, o qual tem demonstrado preservar a sensibilidade do instrumento,^(^ ^14^ ^)^ além de promover uma equivalência apropriada entre as versões. Além disso, sabe-se que a estrutura interna, a semântica e as características psicométricas de um instrumento podem ser alteradas quando esse é traduzido para outra língua. Isso ocorre com maior frequência se a equivalência cultural não for corretamente realizada. A necessidade de se levar em conta as influências culturais na saúde e na doença está progressivamente sendo reconhecida em estudos multicêntricos e internacionais. Quando se adapta um instrumento de avaliação de qualidade de vida, o objetivo é obter medidas de saúde que sejam apropriadas e válidas em diferentes grupos culturais. Isso significa desenvolver um instrumento com equivalência conceitual em diferentes culturas.^(^ ^30^ ^)^

As dificuldades observadas na etapa de tradução para o português decorreram da necessidade de se realizar uma tradução conceitual. Não foram observadas dificuldades na adaptação de palavras referentes a sintomas, atividades físicas e do cotidiano; entretanto, algumas expressões idiomáticas da língua inglesa foram motivo de revisão e discussão, entre elas, "*fed up*" e "*overall enjoyment*". Houve também necessidade de adequação do tempo verbal para que a ação questionada fizesse sentido na língua portuguesa. Na etapa da aprovação pelo autor, apenas dois itens foram questionados quanto a diferenças na tradução literal; entretanto, por manterem a equivalência conceitual, segundo o próprio autor do questionário original, não foram necessárias alterações. Após a aprovação da versão retraduzida, reuniu-se um comitê de especialistas para avaliar a versão em língua portuguesa do questionário com o objetivo de se detectar erros, emitir sugestões e avaliar seu conteúdo e estrutura. Nessa etapa, a participação de membros bilíngues é de valor particular para a composição do comitê de especialistas.^(^ ^14^ ^)^

A primeira modificação realizada foi quanto ao formato do questionário. A versão original utiliza opções de resposta em uma escala Likert em posição sequencial horizontal. A versão foi reformatada como uma tabela horizontal com sete colunas internas em linha única na qual todas as opções foram dispostas em um contínuo crescente. A modificação facilitou a visualização de todas as opções de resposta. Já com o objetivo de se atingir a equivalência semântica, conceitual e idiomática, algumas expressões, palavras, preposições e tempos verbais foram alterados. A dificuldade reside no fato de que não se encontra na língua portuguesa uma palavra que defina literalmente algumas expressões da língua inglesa e, nesse caso, busca-se uma equivalência conceitual para os termos. Correções gramaticais foram efetuadas também pela especialista em português, e o questionário para a aplicação no desdobramento cognitivo foi então confeccionado.

O desdobramento cognitivo (debriefing) é uma etapa fundamental do processo de adaptação cultural, pois mesmo um minucioso processo metodológico não assegura uma tradução equivalente ao instrumento original.^(^ ^14^ ^)^ O questionário foi aplicado a dez participantes para se determinar sua aceitabilidade, clareza e compreensão. Embora os participantes apresentassem uma variabilidade do grau de escolaridade, não foram detectadas dificuldades significativas que impedissem a compreensão do mesmo. Isso demonstra que o instrumento produzido pode ser aplicado às mais variadas classes socioculturais. Optou-se pelo desdobramento item a item para se assegurar que toda a tradução fosse de fácil compreensão, ao invés de se utilizar amostragens randômicas. A análise das respostas oferecidas durante o processo de desdobramento cognitivo demonstrou a necessidade de reavaliação de poucos itens, decorrente da dificuldade de compreensão. Esse achado é de grande relevância, uma vez que mostra a robustez no processo de tradução e de adaptação cultural das etapas iniciais. Assim, a versão final foi formulada após a modificação de três itens, entre eles, o título do questionário, depois de alcançada a unanimidade por acordo entre o comitê revisor e o de especialistas.

Os comentários realizados pelos entrevistados sobre o questionário foram muito positivos. Todos referiram que, de modo geral, o questionário era claro, fácil de entender e de responder, com instruções simples e de rápida aplicação. Foi também considerado de importante relevância na avaliação da tosse crônica, estando bem adaptado a essa condição e abrangendo seus diversos aspectos de forma minuciosa.

Desta forma, o Questionário de Leicester sobre Tosse Crônica encontra-se traduzido e adaptado para a língua portuguesa falada no Brasil. A versão final adaptada para a língua portuguesa mostrou-se simples, de fácil compreensão e aplicação, sendo esse um instrumento único de avaliação das variáveis de qualidade de vida relacionada ao estado de saúde em pacientes portadores de tosse crônica.
